# Effects of microhabitat features on the intraspecific variability of the distribution and functional traits in a highest elevational distributed lizard

**DOI:** 10.1002/ece3.10902

**Published:** 2024-02-15

**Authors:** Da Kang, Chunlin Zhao, Zijian Sun, Guozhu Chen, Jianyi Feng, Wenbo Zhu, Yan Huang, Tian Zhao

**Affiliations:** ^1^ College of Fisheries, Southwest University Chongqing China; ^2^ Yunnan Key Laboratory of Plateau Wetland Conservation, Restoration and Ecological Services Southwest Forestry University Kunming China; ^3^ CAS Key Laboratory of Mountain Ecological Restoration and Bioresource Utilization & Ecological Restoration Biodiversity Conservation Key Laboratory of Sichuan Province Chengdu Institute of Biology, Chinese Academy of Sciences Chengdu China; ^4^ Key Laboratory of Southwest China Wildlife Resources Conservation (Ministry of Education) College of Life Science, China West Normal University Nanchong Sichuan Province China; ^5^ School of Biological and Chemical Engineering (School of Agriculture) Panzhihua University Panzhihua China

**Keywords:** functional morphology, functional niche overlap, functional richness, intraspecific variability, microhabitat variables

## Abstract

Exploring the microhabitat determinants of organisms distribution and functional traits differences can help us better understand the importance of intraspecific variations in ecological niches. Investigations on animals functional niche primarily focused on differences among species and tended to neglect the potential variability within species, despite the fact that the ecological and evolutionary importance of intraspecific variations was widely recognized. In this study, we examined the influence of microhabitat features on the intraspecific variability of the distribution and functional traits of a highest elevational distributed lizard species *Phrynocephalus erythrurus*. To do so, field work was conducted between July and August, 2020 and August and September, 2021 in Namtso watershed in central Xizang, China. Specifically, 11 transects were sampled for *P. erythrurus* individuals, which were measured for a set of 10 morphological traits. Moreover, 11 microhabitat variables that potentially affect the distribution of lizards were also measured for each transect. Our results indicated that juveniles, males, and females exhibited different functional traits, allowing them to occupy distinct functional space. The distribution of juveniles, males, and females was determined by different microhabitat variables such as illuminance and air temperature. More importantly, these variables also determined the intraspecific functional traits variability in this lizard species. All of these results supported previous claims that intraspecific traits variation should be incorporated into functional ecological studies, and diverse microhabitat features should be conserved to maintain high intraspecific diversity. Future studies can focus on the food analysis to explore the linkage between functional traits and resources utilization within animal populations.

## Graphical abstract

This work investigate the effects of microhabitat features on the intraspecific variability of the distribution and functional traits in a highest elevational distributed lizard.

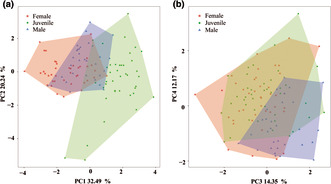

## 1 INTRODUCTION

Understanding species spatial distribution patterns and the related environmental determinants is one of the main concerns in modern ecology (Khatiwada et al., 2019; May et al., 2018). The spatial distribution patterns refer to the differences of organisms composition between habitats, which can be occurred at both interspecific and intraspecific levels. At the interspecific level, different species can occupy distinct habitat due to the determination of environmental variables (Sun, Zhao, et al., 2021; Zhao et al., 2016). Similar situation can also be detected within species. For example, female guppies (*Poecilla reticulata*) usually lived in deep waters, while males preferred shallow waters in rivers of Trinidad (Croft et al., 2009). This is also true for individuals with different developmental stages, as they can exhibit distinct morphological traits, behaviors, and prey preferences during ontogeny (Kingsolver et al., 2011; Zhao et al., 2014). During the past decades, despite quantitative studies have examined the interspecific difference in habitat use of lizards (e.g., Elstrott & Irschick, 2004; Johnson et al., 2008; Ord & Klomp, 2014), little attention has been paid on habitat preference within a lizard species (i.e., between different sexes and stages). Indeed, lizards play important functional roles in ecosystems, regulating the energy flow and nutrient cycling (Miranda, 2017). This is especially true in extreme habitats such as the plateau, where lizards are important consumers maintaining the energy transfer from low to high trophic levels (Jin & Liu, 2010; Sinervo et al., 2010). Therefore, exploring the spatial distribution patterns within a lizard species in a plateau can help us better understand their intraspecific variation in habitat utilization, as well as better conduct species conservation activities in extreme habitats.

In recent decades, with the development of trait‐based ecology, functional traits were considered to be more integrative to understand the relationship between biodiversity and ecosystem functioning (He et al., 2019; Petchey & Gaston, 2006). Functional traits are species' biological features regulating their ecological performance in ecosystems (Smith et al., 2019; Weiss & Ray, 2019). For animals, functional traits are usually represented by their ecomorphological traits, such as fish (Zhao et al., 2019), amphibians (Zhao et al., 2017), lizards (Sinervo et al., 2010), and birds (Carlo et al., 2022). Although these ecomorphological‐based functional traits do not directly measure the functions displayed by animals (e.g., nutrient recycling and trophic interactions), they can effectively reflect and indirectly assess some key functions such as food acquisition, mobility, and defense against predation (Carlo et al., 2022; Sun et al., 2023; Zhao et al., 2019; Zhao, Khatiwada, et al., 2022). In recent decades, some studies indicated that animals ecomorphological‐based functional traits can be affected by habitat features because of phenotypic plasticity (e.g., Shuai et al., 2018; Sun, Zhao, et al., 2021). These studies attempted to reveal the association between environmental conditions and functional traits, which can improve our ability to “predict” the responses of communities to human perturbations (Olden et al., 2002; Pease et al., 2012). However, these studies only focused on the trait variations between species, empirical studies are still needed to investigate the microhabitat determinants of trait variations within species. This is because intraspecific variation of functional traits can also dramatically affect ecosystem functioning (Rudolf & Rasmussen, 2013).

In the present study, a lizard species *Phrynocephalus erythrurus* was selected as the model to investigate the effects of microhabitat conditions on the distribution and functional traits variations within species. Specifically, we first compared the functional traits differences between individuals belonging to different sexes and stages (i.e., juveniles, males, and females). We then explored the microhabitat determinants of the distribution of juveniles, males, and females. Finally, we explored the effects of microhabitat variables on the functional trait variations within species. We proposed the hypothesis that high intraspecific trait variations can be observed within this species, which was affected by microhabitat variables. We also hypothesized that different microhabitat variables determined the distribution of juveniles, males, and females.

## 2 MATERIALS AND METHODS

### 2.1 Study area

**FIGURE 1:**
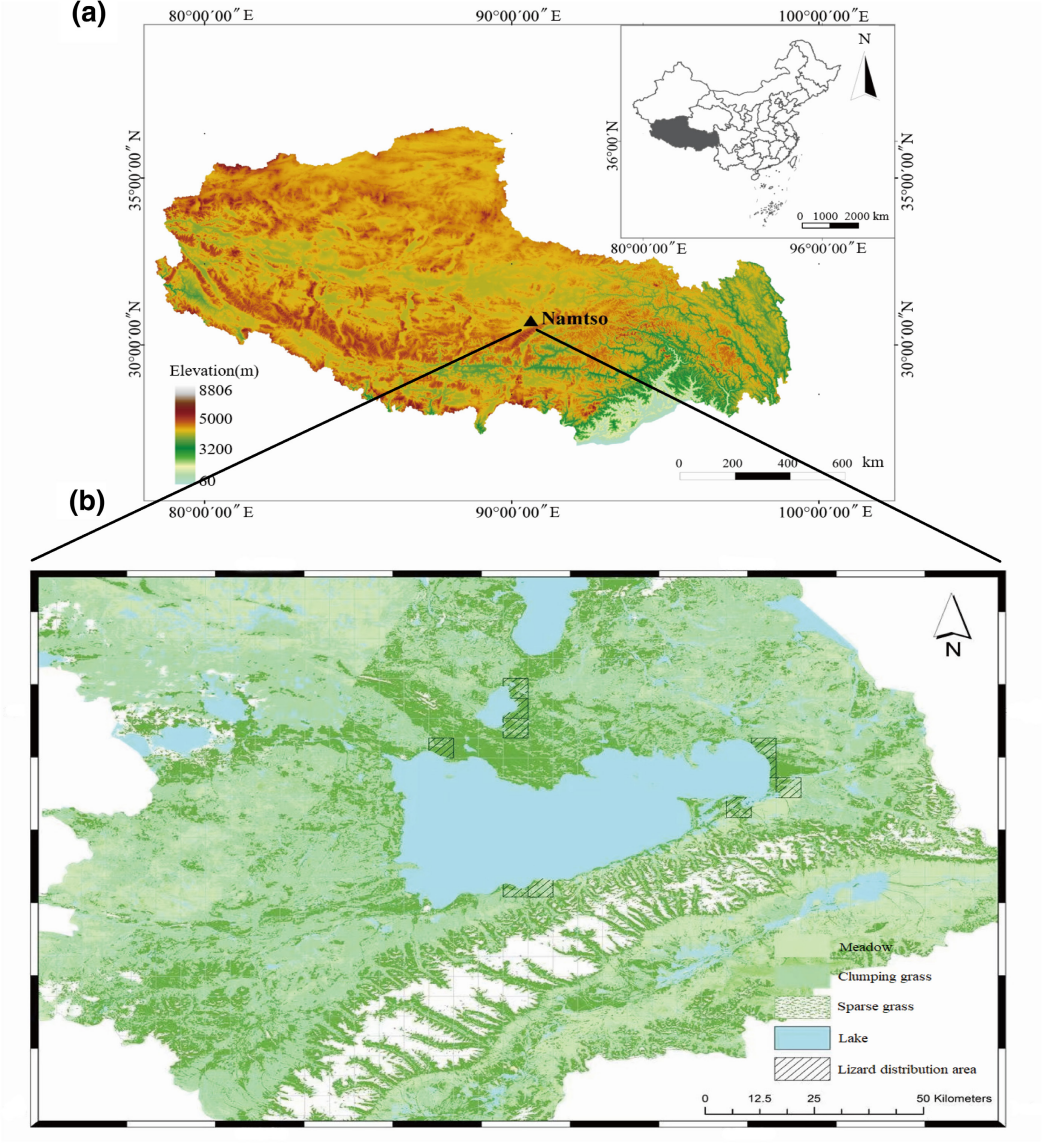
Maps of the study area. (a) The geographical location of Namtso; (b) Overview of the vegetation cover and the lizard distribution area in Namtso watershed (indicated by boxes with dotted black lines).

The present study was conducted in Namtso watershed in central Xizang, China (30°31′–31°09′ N, 90°04′–91°05′ E; Figure 1). This region is located in the semi‐humid and semi‐arid transition zone between Dangxiong and Bange Counties, and belongs to a sub‐frigid plateau monsoon climate. The average annual precipitation is 300–400 mm, and the annual average temperature is −2–0°C (Cong et al., 2009). The average elevation is about 4718 m, and the main vegetation covers are alpine meadows and alpine grasslands (Sun, Guo, et al., 2021).

### 2.2 Model species

*Phrynocephalus erythrurus* is the highest vertically distributed lizard species across the world (Yang et al., 2015). This species is mainly distributed in the Qiangtang Plateau in northern Xizang, with an elevation of 4500–5300 m (Jin & Liu, 2010). Through long‐term natural selection and evolution, *P. erythrurus* has exhibited stable physiological, morphological, and behavioral traits to adapt to extreme plateau environments, such as high capillary density and hemoglobin concentration (Yang et al., 2015), deepened skin color (Porter & Norris, 1969), and viviparous reproduction strategies (Lambert & Wiens, 2013). Previous studies also focused on the relationship between environmental conditions and physiological traits (e.g., metabolic profile; Tang et al., 2013, gut microbiology; Lu et al., 2022) of this species. However, we still need more studies to understand the associations between microhabitats and ecological traits of the species. Based on our previous survey, *P. erythrurus* is the only reptile species in the study area, thus can play an irreplaceable functional role to maintain the stable and health of plateau ecosystems.

### 2.3 Data collection

Based on local environmental conditions, 11 transects (1000 m × 10 m) were constructed and sampled for *P. erythrurus* individuals using visual encounter survey following Peñalver‐Alcázar et al. (2016). This approach involved in two to three people walking along a transect at a slow pace (approximately 1.5 km/h) horizontally and vertically in both directions, searching for all lizard individuals intensively, including the surface ground, rock crevices, and clumps of grass. We also excavated every lizard burrow encountered in the transects to find the hidden individuals using shovels. Sampling was performed between 9:00 and 15:00 in each transect from 31 July to 4 August 2020. To obtain a stronger database, more transects have been sampled from 31 August to 4 September 2021, with three to four transects being sampled per day. We selected this time to do the sampling as *P. erythrurus* is more active during this period in the study area (Gao et al., 2016; Wei et al., 2019; Yao et al., 2000). During the survey, each transect was sampled for three consecutive days to collect a sufficient number of samples. This approach can reduce sampling chance and make the data comparable (Berriozabal‐Islas et al., 2017; Wei et al., 2019). All the lizards encountered were collected by hand (Wishingrad & Thomson, 2019), identified to sex and developmental stages (i.e., juveniles, males, and females), and measured for morphological traits. Based on previous studied, males and females usually exhibited a black and a red coloration of the ventral surface of the tail, respectively. Moreover, the developmental stages can be determined by the snout‐vent length, which was <40 mm for juveniles (Delaney & Warner, 2016; Jin & Liu, 2010; Tang et al., 2013). The captured individuals were injected with passive integrated transponder devices (HONGTENG, GuangZhou, China) for individual identification (Gibbons & Andrews, 2004). After that, all these individuals were released back to their original habitats.

A set of ten morphological traits were measured, including interocular distance (IO), mouth width (MW), head length (HL), head height (HH), head width (HW), forelimb length (FLL), hindlimb length (HLL), tail length (TAL), abdominal maximum width (AMW), and snout‐vent length (SVL; Figure S1). These traits were measured directly using a digital caliper to the nearest 0.01 mm. Moreover, body weight of each individual was measured using a scale to the nearest 0.01 g. All the measurements were conducted by the same person to ensure consistency. Nine eco‐morphological functional traits were obtained based on the calculation of all the above morphological traits (Table 1). These functional traits were unitless values, which can exclude the effects of body size (Kaliontzopoulou et al., 2012). Based on previous studies, these functional traits can reflect two key ecological functions of lizards displayed in ecosystems (i.e., food acquisition and locomotion; Brown et al., 1995; Li et al., 2019; Wang, Wang et al., 2000; Wang, Zheng, 2020; Xu & Ji, 2003). Specifically, mass is related to both food acquisition and locomotion. Moreover, three functional traits reflect lizards food acquisition (i.e., eye position, mouth shape, and head size), while five functional traits describe lizards locomotion (i.e., relative forelimb length, relative hindlimb length, limb ratio, relative tail length, and relative abdominal width).

**TABLE 1:** Nine functional traits of lizards used in this study.

Functional traits	Measure	Ecological meaning
Mass (F/L)	Log (M + 1)	Volume, muscle mass
Eye position (F)	IO/HW	Prey detection
Mouth shape (F)	MW/HW	Size of the prey
Head size (F)	$\sqrt[{3}]{\mathrm{HW}\times \mathrm{HL}\times \mathrm{HH}}$	Food acquisition and bite performance
Relative forelimb length (L)	FLL/SVL	Excavation, acceleration, and maneuverability
Relative hindlimb length (L)	HLL/SVL	Jumping, acceleration, and maneuverability
Limb ratio (L)	FLL/HLL	Coordination, and acceleration
Relative tail length (L)	TAL/SVL	Locomotion balance, and accommodate penis
Relative abdominal width (L)	AMW/SVL	Accommodating embryos

*Note*: The letter in brackets indicates the function associated with each trait (F, food acquisition, and L, locomotion).

We also recorded 11 microhabitat variables after the lizards sampling, including air temperature, air humidity, surface ground temperature, soil temperature, soil humidity, slope, aspect, illuminance, rock coverage, bare soil coverage, clumping grass coverage. These variables were selected based on previous studies showing that they may play important roles in determining lizards distribution (e.g., Delaney & Warner, 2016; Peñalver‐Alcázar et al., 2016; Zeng et al., 2016). Specifically, air temperature and surface ground temperature were measured by digital thermometer. Air humidity was measured by electronic hygrograph. Soil temperature and soil humidity were measured using a soil index detector (DT‐001, China). Slope and aspect were analyzed and calculated by ArcGIS 10.2. Illuminance was measured using a luxmeter (TES1330A, China). Rock coverage, bare soil coverage, and clumping grass coverage were visually estimated in percentage by the same person. Specifically, we estimated the coverage ten times for each transect at ten meters intervals from the starting point, and the average values were used for further analyses (Khatiwada et al., 2019; Peng et al., 2022).

### 2.4 Statistical analyses

We first conducted a principal component analysis (PCA) based on scaled functional traits values of all the individuals (mean of 0 and a standard deviation of 1; Villéger et al., 2008). Axes with eigenvalues >1 were selected to build a multidimensional functional space. Following Mouillot et al. (2013) and Zhao et al. (2014, 2019), three indices were calculated to evaluate the functional traits difference between juveniles, males, and females, including functional identity (FIde), functional richness (FRic), and functional overlap (FOve). FIde represented the abundance weighted averages of functional traits (Mouillot et al., 2013). FRic is the volume of convex hull formed by different groups in the functional space (Villéger et al., 2008). FOve is the overlap of the volume of functional niche between different groups (Villéger et al., 2013). Permutational multivariate analysis of variance (PERMANOVA) was used to test whether there were significant differences in functional identity between juveniles, males, and females (Anderson, 2006). Observed functional richness was calculated as the convex hull volume in the functional space constructed by juveniles, males, and females, respectively. And the observed functional overlap was calculated following Villéger et al. (2013) as follows:

$$\text{FOve}=\frac{\text{FRic}\left(\text{GrpA}⋂\text{GrpB}\right)}{\text{FRic}\left(\text{GrpA}\right)+\text{FRic}\left(\text{GrpB}\right)-\text{FRic}\left(\text{GrpA}⋂\text{GrpB}\right)}.$$

Normally, observed functional richness and functional overlap can be affected by the number of individuals (Zhao et al., 2017). We thus calculated bootstrap functional richness and bootstrap functional overlap by randomly selecting 30, 40, and 41 individuals (i.e., the minimum number of individuals within each group) for each group, respectively. We repeated this procedure with 10,000 times, and calculated the 95% confidence intervals to indicate the significant difference.

We then performed Shapiro test to detect the normality of each microhabitat variable. Spearman's rank correlation tests were used to demonstrate the pairwise correlations between variables that were not normally distributed (i.e., air temperature, soil humidity, illuminance, and rock coverage), while Person correlations were used to test other correlations. Based on our results, surface ground temperature was significantly correlated with soil temperature (*r* = .958, *p* < .001), and bare soil coverage was significantly correlated with clumping grass coverage (*r* = −.907, *p* < .001). We kept soil temperature and clumping grass coverage for further analyses, as they were more important for lizards distribution and activities (Peña‐Joya et al., 2020; Pr et al., 2000). Generalized linear models (GLMs) were constructed to explore the determination of microhabitat variables to the distribution of juveniles, males, and females *P. erythrurus* (Crawley, 2012). In the models, we considered the number of juveniles, males, and females as the dependent variable, respectively, and microhabitat variables were the independent variables. Considering the small sample size and the various combination of microhabitat variables, we compared different GLMs based on the Corrected Akaike Information Criterion (AICc) values (Symonds & Moussalli, 2011). The best model was determined when it exhibited the lowest AICc value, and the difference between the AICc value of the best model (ΔAICc) and the AICc value of the other models was more than 2. Moreover, a model‐averaging was performed (Si et al., 2014; Symonds & Moussalli, 2011). Finally, we used hierarchical partitioning analyses to calculate the relative contributions of different microhabitat variables to the distribution of juveniles, males, and females (Mac Nally, 2002).

To describe the association between lizard functional traits and microhabitat variables, we performed a detrended correspondence analysis (DCA) to determine whether redundancy analysis (RDA; Peters et al., 2016) or canonical correspondence analysis (CCA; Marini et al., 2013) would be the most appreciate model. The DCA ordination gradient was less than 3, suggesting that RDA was the most appropriate model. ANOVA permutation tests (replicated randomly 999 times) were performed to evaluate the RDA model's performance and significance of constraints. In the generated RDA sequencing diagram, the arrow length of microhabitat variables represents the degree of influence on lizard functional traits. The cosine value of the angle between the arrow of microhabitat variables and the arrow of functional traits represents the correlation coefficient between them (Lai et al., 2022).

All the statistical analyses were conducted in R 4.1.0 (R Development Core Team, 2021). Spearman's rank correlation was performed using *psych* package (Revelle, 2013). GLMs were constructed using the *MuMIn* package (Burnham & Anderson, 2004). Hierarchical partitioning was performed using the *vegan* (Oksanen et al., 2013) and *hier.part* packages (Walsh et al., 2004). *FIde*, *FRic*, and *FOve* were calculated based on the *betapart* (Baselga et al., 2013) and *gepmetry* packages (Roussel et al., 2019). In addition, DCA and RDA were conducted using *vegan* package (Oksanen et al., 2013).

## 3 RESULTS

During the two years sampling, a total of 111 *P. erythrurus* individuals were captured, including 40 juveniles, 30 males, and 41 females. The total lengths of juveniles, males, and females were 69.24 mm ± 9.29 (SD), 99.15 mm ± 6.62 (SD), and 98.19 mm ± 6.06 (SD), respectively. The mean body weights of juveniles, males, and females were 2.74 g ± 1.31 (SD), 5.67 g ± 0.98 (SD), and 7.31 g ± 2.04 (SD), respectively.

According to the results of Principal component (PCA), the first four PC axes were kept to build the functional space as their eigenvalues were > 1. These four PC axes accounted for 79.25% of the initial inertia in trait values (PC1 = 32.49%, PC2 = 20.24%, PC3 = 14.35%, PC4 = 12.17%, respectively; Table 2). Specifically, PC1 was mainly driven by mass, head size, and relative hindlimb length. With PC1 values increasing, individuals were more flexible and maneuverable (smaller body weight and head size), associating with a higher endurance (larger relative forelimb and hindlimb) and a wider view (wider interocular distance; Table 2). PC2 was principally associated with limb ratio, relative forelimb length, and relative tail length. With PC2 values increasing, individuals displayed more coordinated limb proportion and longer forelimbs and tail (Table 2).

**TABLE 2:** Pearson correlation coefficients between the four principal components analysis axes and the nine functional traits.

Functional traits	PC1 (32.49%)	PC2 (20.24%)	PC3 (14.35%)	PC4 (12.17%)
*M*	**−0.89**	0.15	0.08	−0.05
EP	**0.60**	−0.14	0.05	**0.36**
MS	**0.37**	**−0.32**	**−0.44**	**−0.43**
HS	**−0.86**	0.16	0.24	−0.16
RFL	**0.54**	**0.79**	−0.04	0.11
RHL	**0.70**	−0.04	**0.61**	−0.04
LR	−0.01	**0.84**	**−0.52**	0.16
RTL	0.10	**0.55**	**0.59**	**−0.34**
RAW	**−0.31**	−0.12	0.20	**0.77**

*Note*: Significant *p* values are in bold.

Abbreviations: EP, eye position; HS, head size; LR, limb ratio; *M*, mass; MS, mouth shape; RAW, relative abdominal width; RFL, relative forelimb length; RHL, relative hindlimb length; RTL, relative tail length.

**FIGURE 2:**
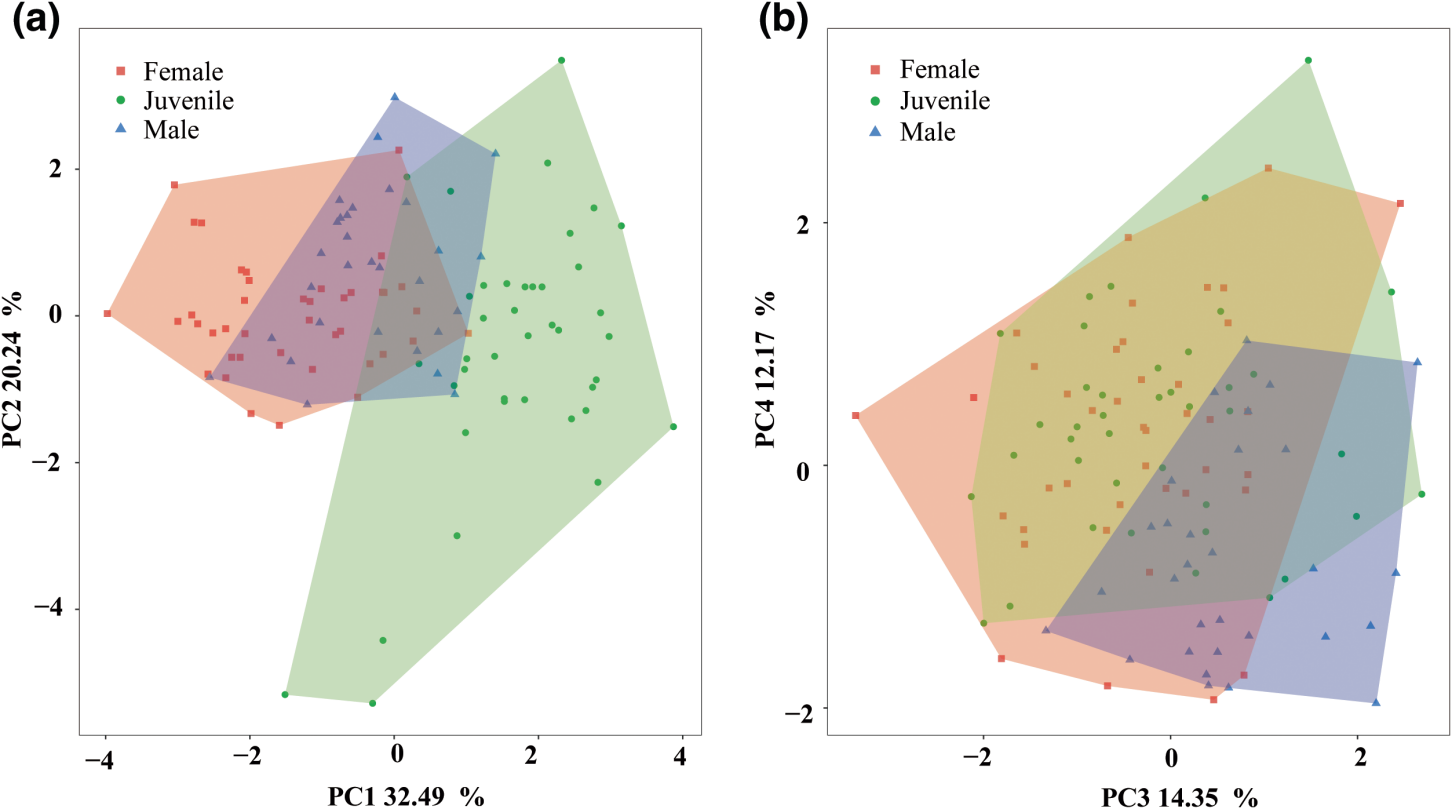
Distribution of different lizard groups (red: female, green: juvenile, blue: male) in the functional spaces. (a) PC1 and PC2 of the functional space; (b) PC3 and PC4 of the functional space. Colored polygons represent the functional richness (convex hull area) of each group.

The position of individuals in the functional space differed significantly among the three lizard groups (PERMANOVA, *p* < .001, Figure 2). Specifically, females were mainly concentrated in the negative area of PC1 (mean = −1.44) and positive area of PC2 (mean = 0.02). Males were mainly concentrated in the negative area of PC1 (mean = −0.30) and positive area of PC2 (mean = 0.28; Figure 2). Juveniles were mainly concentrated in the positive area of PC1 (mean = 1.69) and the negative area of PC2 (mean = −0.49; Figure 2). The observed functional richness of juveniles, males, and females were 30.27%, 5.92%, and 12.43%, respectively. Bootstrap tests revealed that when considering only 30 individuals, the functional richness of females and juveniles were 7.93%–11.79% and 21.70%–26.58%, respectively. Moreover, when considering 40 individuals, the functional richness of females was 12.07%–12.37% (Table 3). Observed functional overlap between females and males was 6.14%, which was 0.51% between females and juveniles, and was 1.92% between males and juveniles. When considering 30 individuals, the functional overlap was 3.55%–4.77% between females and males, and 0.71%–1.16% between males and juveniles. When considering 40 individuals, functional overlap was 0.43%–0.48% between females and males.

**TABLE 3:** Number of individuals in different lizard groups, the observed functional richness, and the bootstrapped functional richness considering 30 and 40 individuals (95% confidence interval).

Groups	*n*	Functional richness		
		Observed	Bootstrapped_*n* = 30_	Bootstrapped_*n* = 40_
Female	41	12.43	7.93%–11.79%	12.07%–12.37%
Male	30	5.92	–	–
Juvenile	40	30.27	21.70%–26.58%	–

**FIGURE 3:**
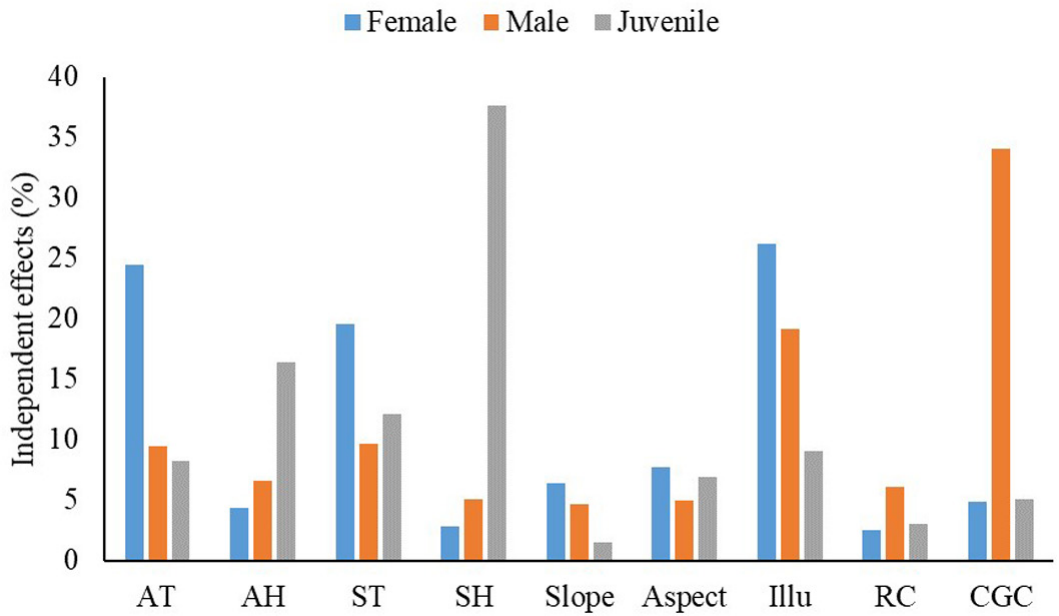
Results of hierarchical partitioning analyses showing the independent contributions of selected microhabitat variables to the distribution of different lizard groups. Details of the abbreviations are in Table 4.

According to the best‐fitted models, we found illuminance had a significant negative effect on both female and male distributions. Clumping grass coverage had a significant negative effect on male distribution (Table 4). Moreover, juvenile distribution was significantly and negatively correlated with air temperature, air humidity, and soil humidity, but was significantly and positively correlated with soil temperature and rock coverage (Table 4). Regarding the independent contribution, hierarchical partitioning analyses indicated that the distribution of females was best explained by illuminance (26.18%), followed by air temperature (24.45%; Figure 3). Clumping grass coverage and illuminance explained 34.06% and 19.21% of the distribution of males, respectively (Figure 3). Finally, soil humidity (37.64%) was the most important contributor to the habitat preference of juveniles, followed by air humidity (16.38%), soil temperature (12.11%), air temperature (8.27%), and rock coverage (3.00%; Figure 3).

**TABLE 4:** The best‐fitted model selected by the generalized linear models (GLMs) for each lizard group.

Group	Intercept	AT	AH	ST	SH	Slope	Aspect	Illu	RC	CGC
Female										
Estimate	4.357	8.293	1.541	−7.195	−0.128	−3.845	1.992	−3.645	0.914	−0.656
SE	1.412	7.454	2.963	5.244	0.969	2.325	1.018	1.551	0.915	0.968
*p*	**.002**	.266	.603	.170	.95	.098	.050	**.019**	.318	.498
Male										
Estimate	3.892	7.560	1.142	−3.138	0.345	−2.411	0.973	−2.686	−1.828	−2.801
SE	1.205	5.798	2.599	3.600	1.111	2.111	0.991	1.253	1.319	1.111
*p*	**.001**	.192	.660	.383	.756	.253	.327	**.032**	.166	**.012**
Juvenile										
Estimate	9.821	−25.812	−16.369	25.155	−10.798	−1.485	2.478	−5.654	5.991	3.897
SE	3.475	13.040	5.385	8.760	4.162	6.833	2.748	2.962	2.545	2.605
*p*	**.005**	**.048**	**.002**	**.004**	**<.001**	.828	.367	.056	**.019**	.135

*Note*: Significant *p* values are in bold.

Abbreviations: AH, air humidity; AT, air temperature; CGC, clumping grass coverage; Illu, illuminance; RC, rock coverage; SH, soil humidity; ST, soil temperature.

**FIGURE 4:**
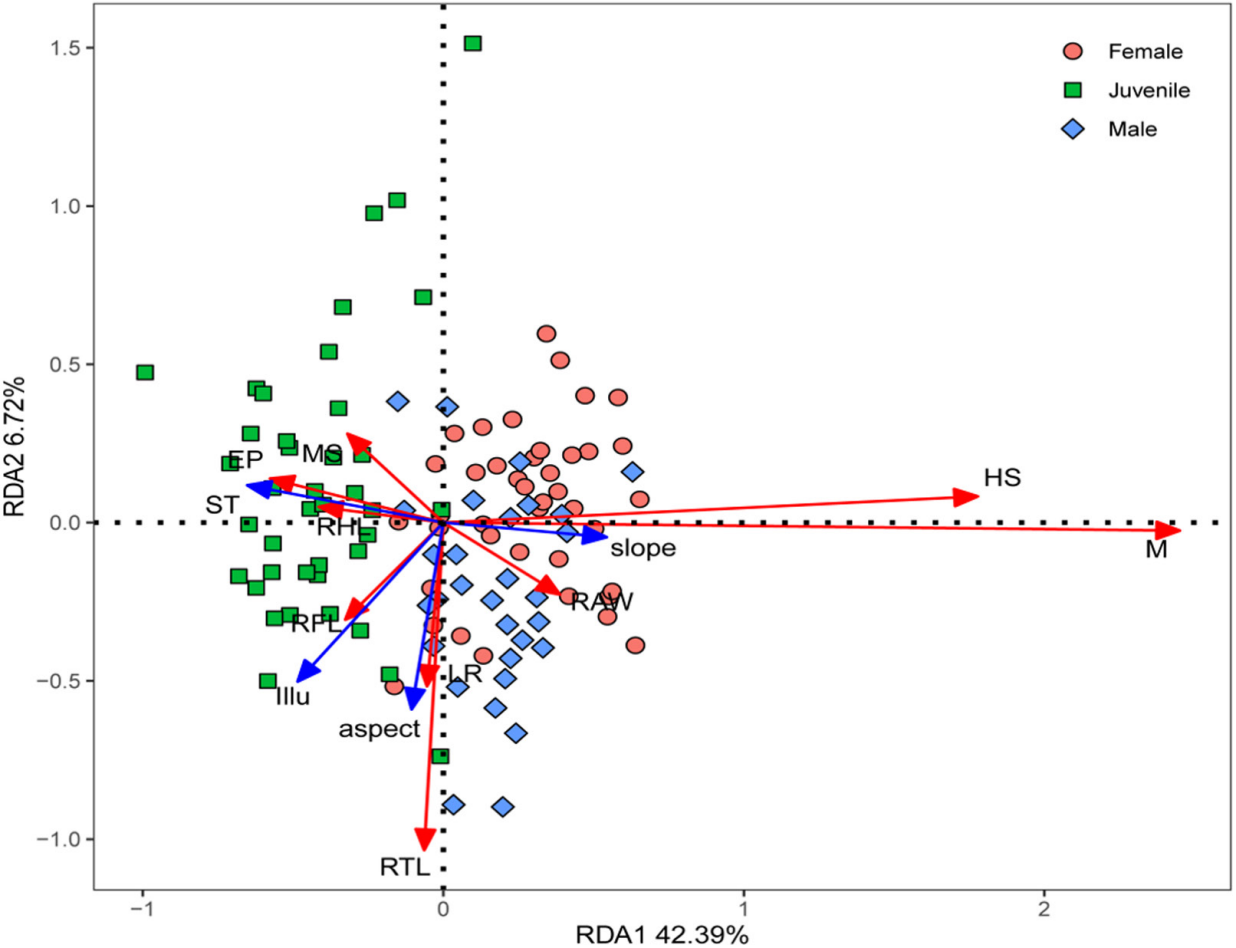
Redundancy analysis triplot showing the relationships between lizards functional traits and microhabitat variables. The RDA triplot only retains the significant microhabitat variables (*p* < .05) which are represented by blue lines. Red lines indicate the functional traits. Red circles, green squares, and blue rhombus represent the location of different lizard groups in the functional space, respectively. Details of the abbreviations are in Table 2.

The RDA model revealed the relationships between functional traits and microhabitat variables were significant (*p* = .002; Figure 4). The combined effects of the first two canonical axes explain 49.11% of the total variation (42.39% and 6.72%, respectively). Specifically, mouth shape, eye position, and relative hindlimb length were positively correlated with soil temperature, and were negatively correlated with slope. Head size, mass, and relative abdominal width were positively correlated with slope, and were negatively correlated with soil temperature. Limb ratio and relative tail length were positively correlated with aspect. Relative forelimb length was positively correlated with illuminance (Figure 4).

## 4 DISCUSSION

The present study demonstrated that juveniles, males, and females of *P. erythrurus* exhibited different functional traits, leading to the occupation of distinct functional niche. This result is consistent with previous observations showing that high intraspecific variability in functional traits was widely existed in animal populations (Rudolf & Rasmussen, 2013; Zhao et al., 2014). Specifically, females and males showed stronger torsos, lager head sizes, and stubby tails, associating with increased fecundity and reproductive output (Liang et al., 2021; Shine, 1988; Toyama et al., 2022). Juveniles performed slender limbs and tails, and wider interocular distances, thus they can move more flexibly and have a wide field of vision, leading them better able to avoid predators (Irschick et al., 1996; Macrini et al., 2003). These traits variations can be attributed to ontogenetic shift, associating with the change in food resources (Bolnick et al., 2011; Moran et al., 2016), and more importantly, the specific microhabitat preference (Buckley et al., 2010). Specifically, juveniles were mainly distributed in microhabitats with higher soil temperature and rock coverage, but lower soil and air humidity. Previous studies indicated that higher soil temperature can help juveniles to maintain their body temperature, and thus the normal metabolism (Huey & Kingsolver, 1989; Ohlberger, 2013). Moreover, these environmental conditions can provide juveniles with sufficient food resources such as beetles and spiders (Maia‐Carneiro et al., 2017; Sutherland, 2011). Abundant rocks were associated with sufficient refuges for juveniles, reducing the risk of being predation. Furthermore, both males and females preferred low‐illuminant conditions, as high illuminance meant high‐ultraviolet radiation in the plateau, which may affect their survival (Clusella‐Trullas & Chown, 2014; Solomon, 2008). Males also preferred microhabitats with lower clumping grass coverage, because they were usually more pioneering and had a stronger sense of territoriality in the population (Zhao, Feng, et al., 2022), promoting the conspicuousness of sexual communication (Hedrick, 2000; Husak et al., 2006).

Intraspecific functional trait variability was also determined by microhabitat variables. Specifically, lizards with larger head size, mass, and relative abdominal width were usually detected in microhabitat with higher slope and lower soil temperature. These kinds of lizards exhibited a stronger ability for predation and movement and thus can help them better utilize the shelters and food resources (Damme et al., 1995). For instance, Herrel et al. (2001) indicated that larger head size can help lizards to better hunt for prey in microhabitat with higher slope, and Verwaijen et al. (2002) demonstrated that stronger body allowed lizards to better adapt to the complex terrain with higher slope. More importantly, these lizards can store more heat to adapt to cold temperature in these habitats, as these traits are associated with energy storage (Ortega et al., 2016). We also found that lizards with larger mouth shape, eye position, and relative hindlimb length were usually detected in microhabitat with lower slope and higher soil temperatures (Slavenko et al., 2021). This is because lizards under such environmental conditions require a high level of mobility and wide field view so that they can prey on food and avoid predators more easily (Melville & Swain, 2000). In addition, we found that lizards with larger limb ratios and relative tail lengths were usually detected in microhabitats with higher aspects, and lizards with larger relative forelimb length were correlated with higher illuminance. Aspect reflected the duration of solar radiation, and illuminance reflected the intensity of solar radiation. Larger limb ratio, relative tail length, and relative forelimb length indicated a larger surface area‐to‐volume ratio in lizards (Carothers et al., 1997). In high elevations, low‐ambient temperatures lead to short windows available for activity (Monasterio et al., 2009). Therefore, such trait variations allowed lizards to acquire thermal energy more efficiently (Slavenko et al., 2021).

## 5 CONCLUSIONS

In conclusion, the present study demonstrated a significant variation in functional traits within a lizard species (i.e., between juveniles, males, and females). Meanwhile, different microhabitat variables have significant effects on the distribution of juveniles, males, and females *P. erythrurus*. Importantly, microhabitat variables also affect the variability of functional traits, leading to distinct functional roles within populations. All of these results supported previous claims that intraspecific traits variation should be incorporated into functional ecological studies, and diverse microhabitat features should be conserved to maintain high intraspecific diversity. However, since this study was only conducted during a specific time period that coincides with the cessation of mating, future studies should consider the non‐breeding seasons to better understand the intraspecific microhabitat preferences for this species. In addition, food analysis could be conducted to explore the linkage between functional traits and resources utilization within animal populations.

## AUTHOR CONTRIBUTIONS

**Da Kang:** Data curation (lead); formal analysis (lead); investigation (equal); writing – original draft (lead). **Chunlin Zhao:** Data curation (equal); investigation (equal). **Zijian Sun:** Resources (equal); validation (equal). **Guozhu Chen:** Funding acquisition (equal); validation (equal); writing – review and editing (equal). **Jianyi Feng:** Investigation (equal); resources (equal). **Wenbo Zhu:** Data curation (equal); investigation (equal); resources (equal). **Yan Huang:** Writing – review and editing (equal). **Tian Zhao:** Funding acquisition (lead); investigation (lead); methodology (lead); supervision (lead); visualization (lead); writing – review and editing (lead).

## CONFLICT OF INTEREST STATEMENT

The authors declare that they have no competing or conflict of interest.

## Supporting information


Figure S1.

## Data Availability

Data used in this study can be found in Figshare (https://figshare.com/s/181026ca2345042ccd62).
