# Supplementary material for: Effects of microhabitat features on the intraspecific variability of the distribution and functional traits in a highest elevational distributed lizard
Source: Ecol Evol. 2024 Feb 15;14(2):e10902. doi: 10.1002/ece3.10902 (PMC10869896; doi:10.1002/ece3.10902)
Supplement: Supplementary file 1 — Figure S1. [file ECE3-14-e10902-s001.docx]

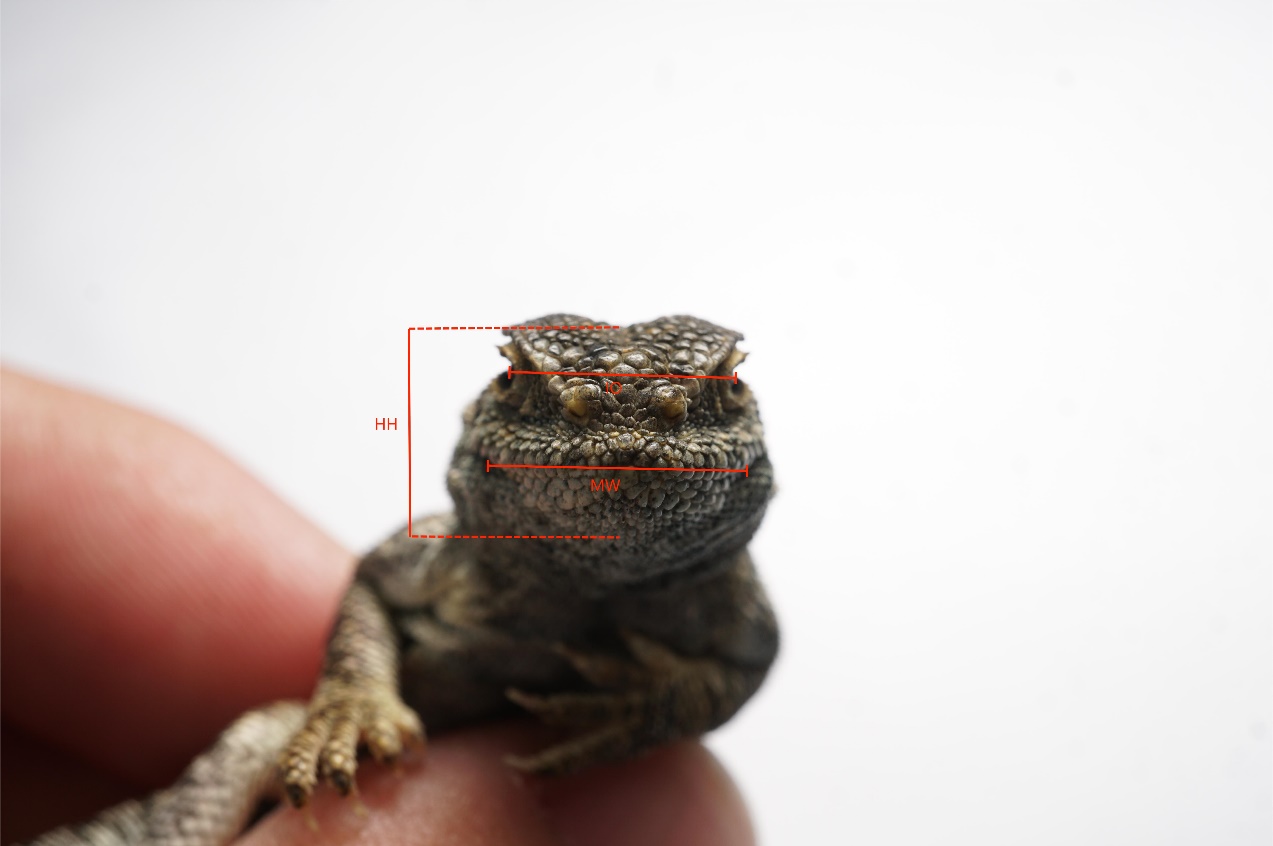


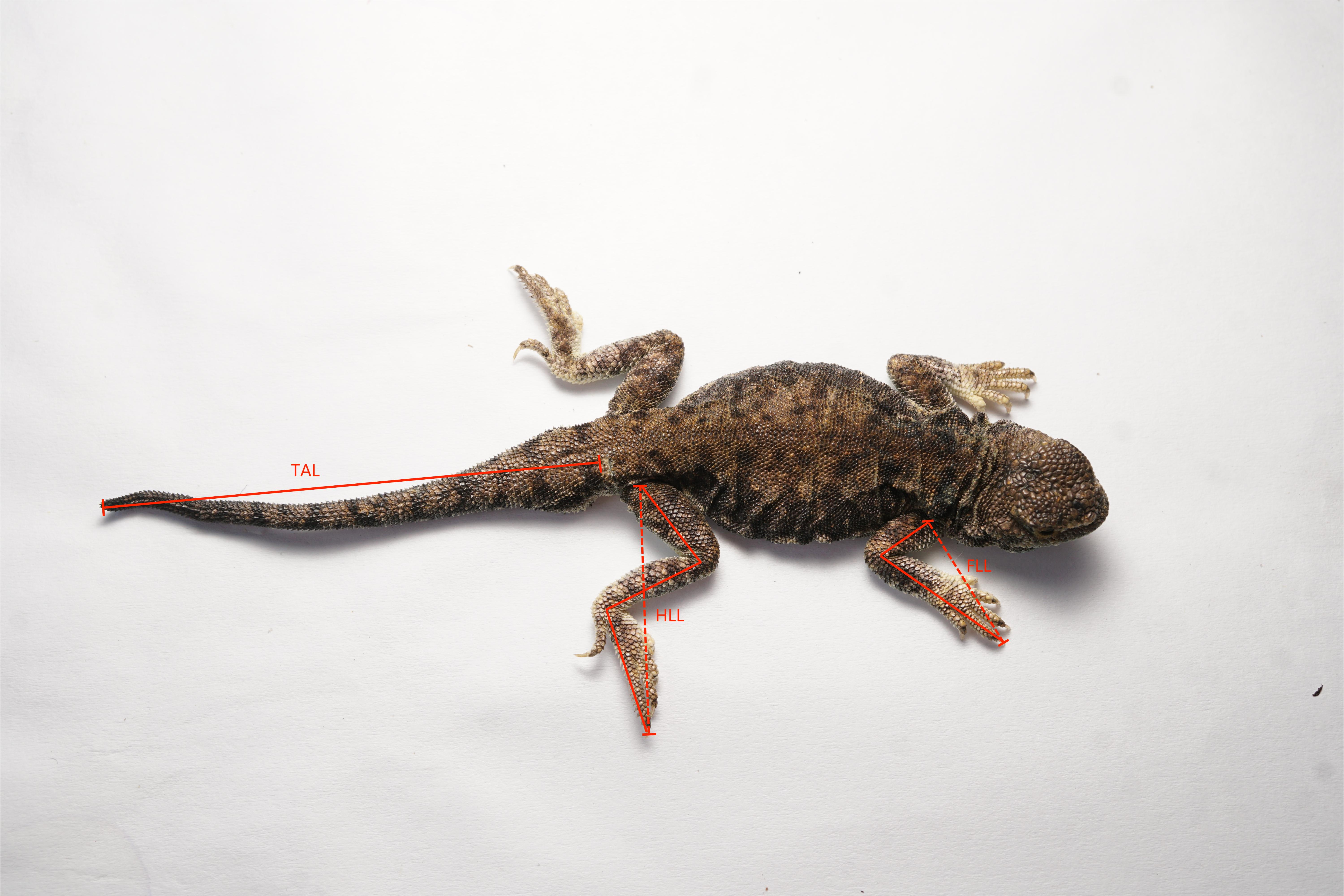

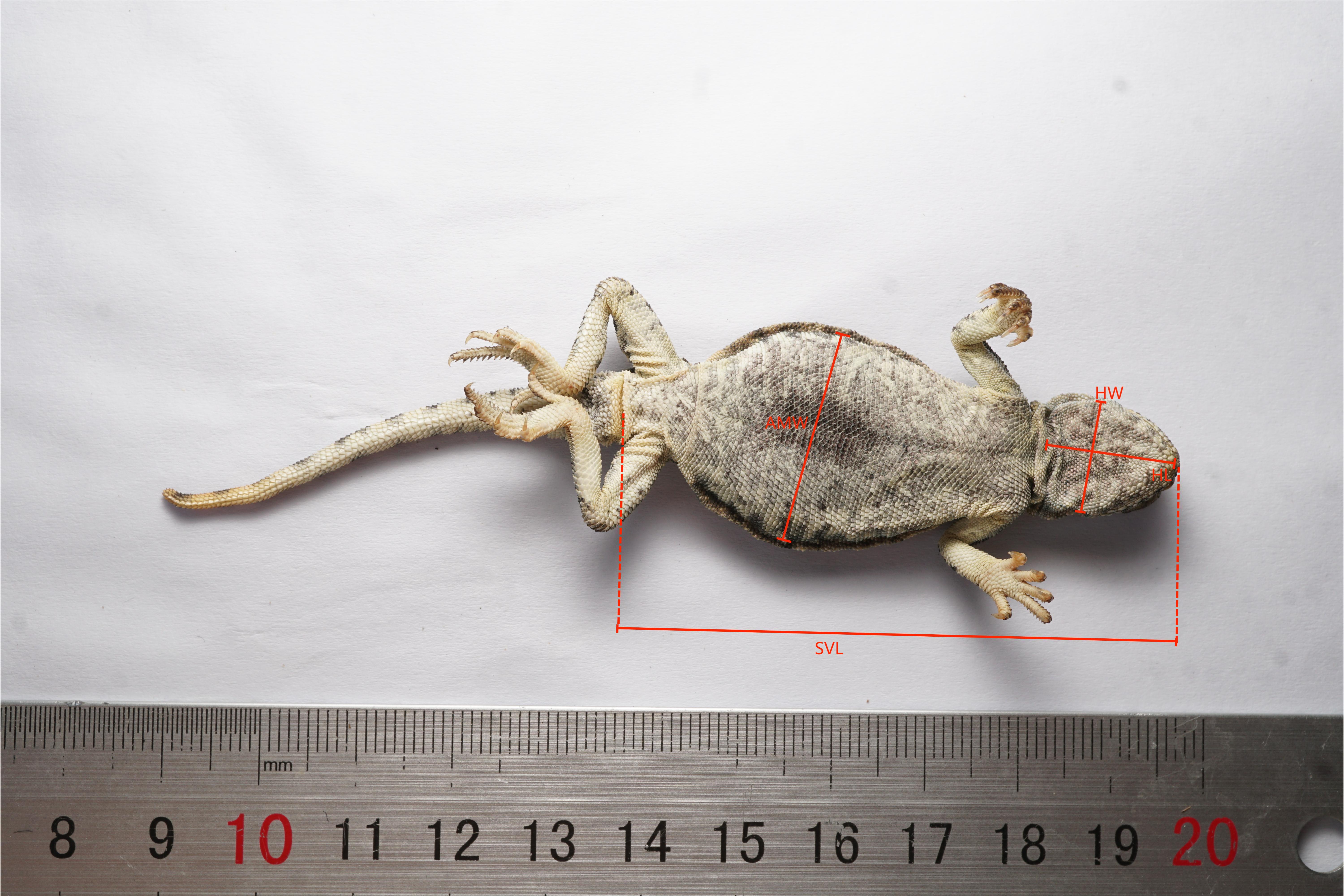


Figure S1 The measurement of morphological traits of lizards. Details of abbreviations are as follows: IO, interoculaer distance; MW, mouth width; HL, head length; HH, head height; HW, head width; FLL, forelimb length; HLL, hindlimb length; TAL, tail length; AMW, abdominal maximum width; SVL, snout-vent length.
